# Complete genome sequence of *Desulfotomaculum acetoxidans* type strain (5575^T^)

**DOI:** 10.4056/sigs.39508

**Published:** 2009-11-22

**Authors:** Stefan Spring, Alla Lapidus, Maren Schröder, Dorothea Gleim, David Sims, Linda Meincke, Tijana Glavina Del Rio, Hope Tice, Alex Copeland, Jan-Fang Cheng, Susan Lucas, Feng Chen, Matt Nolan, David Bruce, Lynne Goodwin, Sam Pitluck, Natalia Ivanova, Konstantinos Mavromatis, Natalia Mikhailova, Amrita Pati, Amy Chen, Krishna Palaniappan, Miriam Land, Loren Hauser, Yun-Juan Chang, Cynthia D. Jeffries, Patrick Chain, Elizabeth Saunders, Thomas Brettin, John C. Detter, Markus Göker, Jim Bristow, Jonathan A. Eisen, Victor Markowitz, Philip Hugenholtz, Nikos C Kyrpides, Hans-Peter Klenk, Cliff Han

**Affiliations:** 1 DSMZ - German Collection of Microorganisms and Cell Cultures GmbH, Braunschweig, Germany; 2 DOE Joint Genome Institute, Walnut Creek, California, USA; 3 Los Alamos National Laboratory, Bioscience Division, Los Alamos, New Mexico, USA; 4 Biological Data Management and Technology Center, Lawrence Berkeley National Laboratory, Berkeley, California, USA; 5 Oak Ridge National Laboratory, Oak Ridge, Tennessee, USA; 6 Lawrence Livermore National Laboratory, Livermore, California, USA; 7 University of California Davis Genome Center, Davis, California, USA; * **Corresponding author**: Hans-Peter Klenk

**Keywords:** sulfate-reducer, hydrogen sulfide, piggery waste, mesophile, motile, sporulating, obligate anaerobic, *Peptococcaceae*, *Clostridiales*, *Firmicutes*

## Abstract

*Desulfotomaculum acetoxidans* Widdel and Pfennig 1977 was one of the first sulfate-reducing bacteria known to grow with acetate as sole energy and carbon source. It is able to oxidize substrates completely to carbon dioxide with sulfate as the electron acceptor, which is reduced to hydrogen sulfide. All available data about this species are based on strain 5575^T^, isolated from piggery waste in Germany. Here we describe the features of this organism, together with the complete genome sequence and annotation. This is the first completed genome sequence of a *Desulfotomaculum* species with validly published name. The 4,545,624 bp long single replicon genome with its 4370 protein-coding and 100 RNA genes is a part of the *** G****enomic* *** E****ncyclopedia of* *** B****acteria and* *** A****rchaea * project.

## Introduction

Strain 5575^T^, also known as “Göttingen” strain (= DSM 771 = ATCC 49208 = VKM B-1644 = KCTC 5769) is the type strain of the species *Desulfotomaculum acetoxidans* [1]. Strain 5575^T^ is the only strain of the species that is available from public culture collections. It was isolated from piggery waste in Göttingen, Germany. Widdel and Pfennig [2] reported the isolation of additional strains from animal manure, rumen content and dung-contaminated freshwater habitats and concluded that members of *D. acetoxidans* are primarily intestinal bacteria. Unclassified strains with rather high 16S rRNA gene sequence similarity to strain 5575^T^ were reported from rice field soil (AJ012600 and AJ012601, 98%) and from a freshwater sediment in The Netherlands [3]. A complete genome sequence of the strain ‘*Desulfotomaculum reducens*’ MI-1 was recently determined by the DOE Joint Genome Institute (GenBank accession number NC_009253). However, this strain is only distantly related with *D. acetoxidans* 5575^T^, both sharing a 16S rRNA sequence similarity of only 86%, and has no taxonomic status, because the species epithet was never validly published. Here we present a summary classification and a set of features for *D. acetoxidans* strain 5575^T^ together with the description of the complete genomic sequencing and annotation.

## Classification and features

The genus *Desulfotomaculum* currently represents a rather heterogeneous taxon. The available 16S rRNA gene sequences (GenBank accession numbers AB294139 and NR_026409) of the type strain of *Desulfotomaculum guttoideum*, DSM 4024, appear to be unrelated to the type species of the genus, but show high similarity to *Clostridium sphenoides* and *C. celerescens*, which both belong to cluster XIVa of the clostridia [4]. An investigation of the phenotypic traits of *D. guttoideum* strain DSM 4024 indicated its affiliation to the species *C. sphenoides*, which was also confirmed by a DNA-DNA reassociation value above 70% with the type strain of *C. sphenoides* (unpublished results). This indicates that either the published species description of *D. guttoideum* is erroneous or the originally described strain is not identical with the culture that was deposited in culture collections. Apart from this species, the genus *Desulfotomaculum* is paraphyletic and comprises several distinct phenotypic types including mesophilic species, like *D. acetoxidans*, moderate thermophiles (e.g. *D. thermosubterraneum*), halophiles (*D. halophilum*) and alkaliphiles (*D. alkaliphilum*). The type species of the genus, *D. nigrificans*, is a moderate thermophile and shares only 85% 16S rRNA gene sequence similarity with *D. acetoxidans*, indicating that the latter species might have been misclassified. The members of the genus *Desulfotomaculum* are affiliated to the family *Peptococcaceae* of the order *Clostridiales*, within the phylum *Firmicutes*.

**Figure 1:**
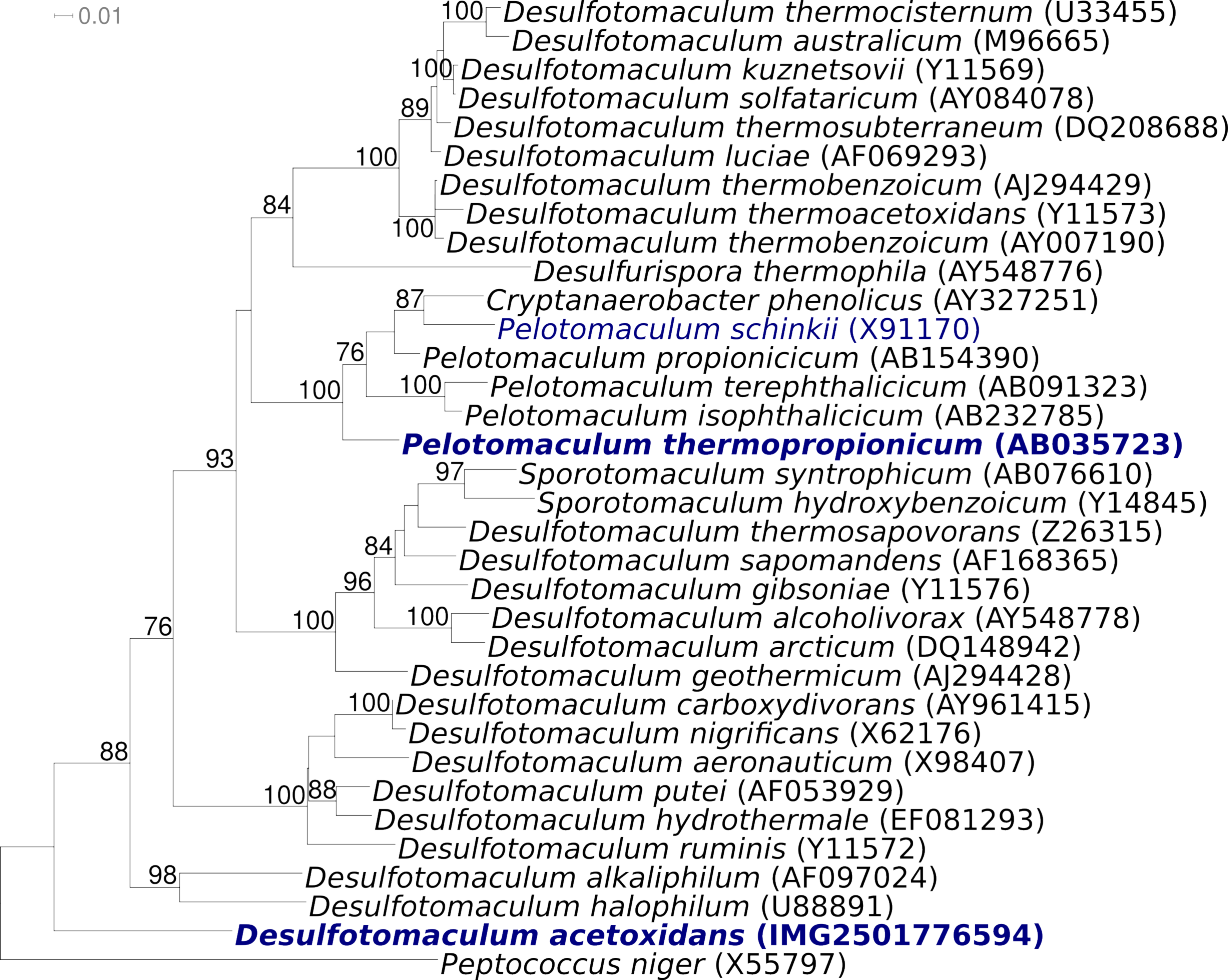
Phylogenetic tree highlighting the position of *D. acetoxidans* strain 5575^T^ relative to all type strains of the genus *Desulfotomaculum*, and the closely related genera *Cryptanaerobacter*, *Desulfurispora*, *Pelotomaculum* and *Sporotomaculum*, which appear to be nested within the paraphyletic genus *Desulfotomaculum*. The tree was inferred from 1294 aligned characters [5,6] of the 16S rRNA sequence under the maximum likelihood criterion [7] and rooted with the type strain of the type species of the family *Peptococcaceae*. The branches are scaled in terms of the expected number of substitutions per site. Numbers above branches are support values from 1000 bootstrap replicates if larger than 60%. Lineages with type strain genome sequencing projects registered in GOLD [8] are shown in blue, published genomes in bold.

Figure 1 shows the phylogenetic neighborhood of *D. acetoxidans* strain 5575^T^ in a 16S rRNA based tree. The ten 16S rRNA gene copies in the genome of strain 5575^T^ differ by up to 41 nucleotides (2.6%) from each other, and by up to 35 nucleotides (2.2%) from the previously published 16S rRNA sequence generated from DSM 771 (Y11566). *D. guttoideum*, DSM 4024, has not been included in the phylogenetic analysis for reasons given above.

**Figure 2:**
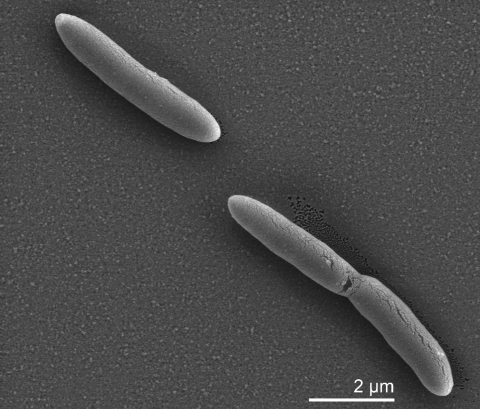
Scanning electron micrograph of vegetative cells of *D. acetoxidans* strain 5575^T^ (Manfred Rohde, Helmholtz Centre for Infection Research, Braunschweig)

Vegetative cells of *D. acetoxidans* 5575^T^ are straight to slightly curved rods with pointed ends and dimensions of 1.0-1.5 µm ×3.5-9.0 µm (Table 1 and Figure 2). Motility is conferred by a single polar flagellum [1]. Cells were originally described to stain Gram-negative [1], which is a typical trait among *Desulfotomaculum* species, but all *Desulfotomaculum* strains examined so far by electron microscopy have a cell wall structure of the Gram-positive type [20]. Spherical spores of 1.5 µm diameter are located in a subterminal position and cause a swelling of cells resulting in a typical spindle shaped morphology. Spores are preferentially formed in agar colonies upon prolonged incubation with acetate as substrate. Formation of spores is often accompanied by the production of gas vacuoles that appear as conic refractile areas adjacent to the spores in sporulating mother cells. Growth occurs between 20 and 40°C with an optimum at 36°C. The pH range for growth is 6.6–7.6, with an optimum at 7.1 [1]. The salinity optimum for growth of *D. acetoxidans* is 1 g/l NaCl and growth is inhibited above 7 g/l NaCl, which is typical for strains showing an adaptation to freshwater habitats [2].

**Table 1:** Classification and general features of *D. acetoxidans* strain 5575^T^ in accordance with the MIGS recommendations [9]

**MIGS ID**	**Property**	**Term**	**Evidence code**	
	Current classification	Domain *Bacteria*		TAS [10]
		Phylum *Firmicutes*		TAS [11]
		Class *Clostridia*		TAS [11]
		Order *Clostridiales*		TAS [12]
		Family *Peptococcaceae*		TAS [13,14]
		Genus *Desulfotomaculum*		TAS [14-16]
		Species *Desulfotomaculum acetoxidans*		TAS [1]
		Type strain 5575		TAS [1]
	Gram stain	negative		TAS [1]
	Cell shape	rod with pointed ends		TAS [1]
	Motility	motile (single polar flagellum)		TAS [1]
	Sporulation	spherical endospores		TAS [1]
	Temperature range	20-40°C		TAS [1]
	Optimum temperature	36°C		TAS [1]
	Salinity	1-7 g/l		TAS [2]
MIGS-22	Oxygen requirement	obligate anaerobic		TAS [1]
	Carbon source	CO_2_, acetate		TAS [1,17]
	Energy source	H_2_, acetate, n-butyrate, ethanol, n-butanol		TAS [2,17]
MIGS-6	Habitat	animal intestinal microflora, fresh water, mud, sea water sediment, soil		TAS [2]
MIGS-15	Biotic relationship	free living		
MIGS-14	Pathogenicity	none		TAS [18]
	Biosafety level	1		TAS [18]
	Isolation	piggery waste		TAS [1]
MIGS-4	Geographic location	Göttingen, Germany		NAS
MIGS-5	Sample collection time	1976		NAS
MIGS-4.1 MIGS-4.2	Latitude – Longitude	+51.54 - +9.93		NAS
MIGS-4.3	Depth	not reported		
MIGS-4.4	Altitude	240 m		NAS

Evidence codes - IDA: Inferred from Direct Assay (first time in publication); TAS: Traceable Author Statement (i.e., a direct report exists in the literature); NAS: Non-traceable Author Statement (i.e., not directly observed for the living, isolated sample, but based on a generally accepted property for the species, or anecdotal evidence). These evidence codes are from the Gene Ontology project [19]. If the evidence code is IDA, then the property was observed for a living isolate by one of the authors or an expert mentioned in the acknowledgments.

Substrates allowing good growth were found to be acetate and butyrate, whereas long chain fatty acids or carbohydrates were not utilized [2]. In addition, ethanol, n-butanol, iso-butyrate and n-valerate were identified as suitable substrates. With acetate as substrate, only sulfate was reported to be used as electron acceptor, but not sulfite, thiosulfate or fumarate [2]. No fermentative growth on organic substrates in media without sulfate was observed [1]. Biotin was identified as sole growth factor in defined media [2].

### Chemotaxonomy

Redox difference spectra indicate the presence of membrane bound *b*-type cytochromes, whereas no cytochromes *c* or soluble cytochromes were detected. The CO-difference spectrum of the soluble cell fraction revealed presence of a dissimilatory sulfite reductase of the type P582 [1,2]. *D. acetoxidans* contains only menaquinones, mainly of the MK-7 type and small amounts of MK-6 [21]. Dowling *et al*. [22] determined the whole-cell fatty acid pattern of *D. acetoxidans* strain 5575^T^ and found a dominance of straight-chain, even-numbered fatty acids, whereas neither 10-methyl nor cyclopropyl fatty acids were present. The predominant fatty acids this organism were 16:0 (34.0%), 16:1 ω7c (24.4%) and 18:1 ω7c (24.1%), followed by 16:1 ω9 (5.9%) and 16:1 ω5 (4.8%). The abundance of distinct fatty acids in this species apparently depends strongly on the medium composition: It was found that supplementation of the growth medium with volatile fatty acids led to a decreased proportion of even-numbered fatty acids from 99 to 67% [22]; in addition, Londry *et al*. reported that under conditions of autotrophic growth the proportion of 16:1 fatty acids decreased, whereas 18:1 fatty acids increased compared to growth on acetate as carbon source [23].

## Genome sequencing and annotation

### Genome project history

This organism was selected for sequencing on the basis of its phylogenetic position, and is part of the *** G****enomic* *** E****ncyclopedia of* *** B****acteria and* *** A****rchaea * project. The genome project is deposited in the Genomes OnLine Database [8] and the complete genome sequence in GenBank. Sequencing, finishing and annotation were performed by the DOE Joint Genome Institute (JGI). A summary of the project information is shown in Table 2.

**Table 2:** Genome sequencing project information

**MIGS ID**	**Property**	**Term**
MIGS-31	Finishing quality	Finished
MIGS-28	Libraries used	Two genomic libraries - 8 kb pMCL200 and fosmid pcc1Fos
MIGS-29	Sequencing platforms	ABI3730
MIGS-31.2	Sequencing coverage	8.56x Sanger
MIGS-30	Assemblers	phrap
MIGS-32	Gene calling method	Prodigal, GenePRIMP
	GenBank ID	CP001720
	GenBank Date of Release	September 10, 2009
	GOLD ID	Gc01106
	NCBI project ID	27947
	Database: IMG-GEBA	2501651223
MIGS-13	Source material identifier	DSM 771
	Project relevance	Tree of Life, GEBA

### Growth conditions and DNA isolation

*D. acetoxidans* strain 5575^T^, DSM 771, was grown anaerobically in DSMZ medium 124 [24]at 37°C. DNA was isolated from 1-1.5 g of cell paste using Qiagen Genomic 500 DNA Kit (Qiagen, Hilden, Germany) following the manufacturer's instructions, with a modified protocol for cell lysis (modification LALMP), as described in [25].

### Genome sequencing and assembly

The genome of *D. acetoxidans* strain 5575^T^ was sequenced using a combination of 8 kb and fosmid genomic libraries on a Sanger sequencing platform. The Phred/Phrap/Consed software package (http://www.phrap.com) was used for sequence assembly and quality assessment. Possible mis-assemblies were corrected with Dupfinisher or transposon bombing of bridging clones [26]. Gaps between contigs were closed by editing in Consed, custom primer walk or PCR amplification. A total of 3,281 Sanger finishing reads were produced to close gaps, to resolve repetitive regions, and to raise the quality of the finished sequence. The error rate of the completed genome sequence is less than 1 in 100,000. Together all sequence types provided 9.2× coverage of the genome. The completed genome sequences of *D. acetoxidans* contains 46,605 reads.

### Genome annotation

Genes were identified using Prodigal [27] as part of the Oak Ridge National Laboratory genome annotation pipeline, followed by a round of manual curation using the JGI GenePRIMP pipeline [28]. The predicted CDSs were translated and used to search the National Center for Biotechnology Information (NCBI) nonredundant database, UniProt, TIGRFam, Pfam, PRIAM, KEGG, COG, and InterPro databases. Additional gene prediction analysis and functional annotation was performed within the Integrated Microbial Genomes Expert Review (IMG-ER) platform [29].

### Genome properties

**Figure 3:**
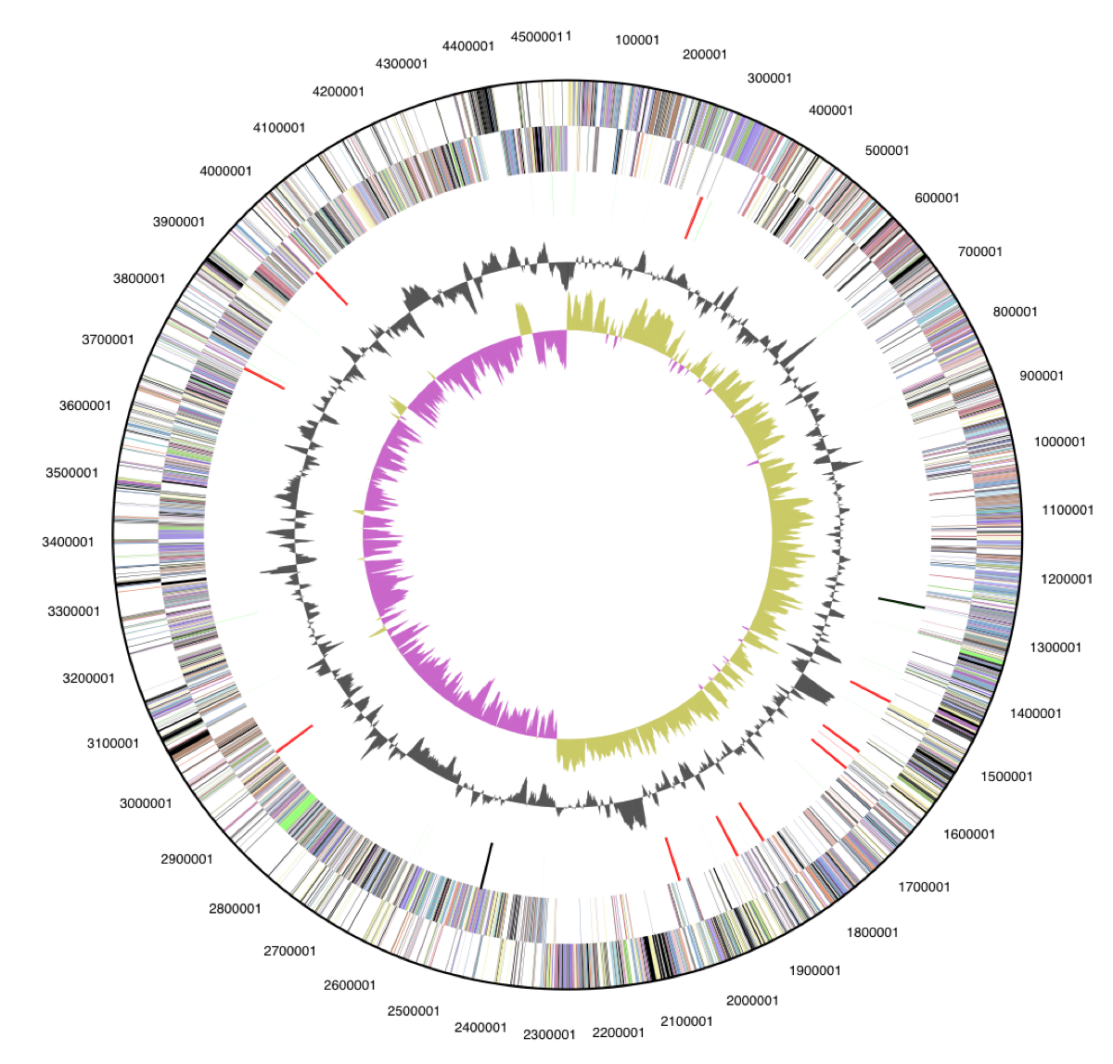
Graphical circular map of the genome. From outside to the center: Genes on forward strand (color by COG categories), Genes on reverse strand (color by COG categories), RNA genes (tRNAs green, rRNAs red, other RNAs black), GC content, GC skew.

The genome is 4,545,624 bp long with a 41.6% GC content (Table 3 and Figure 3). Of the 4470 genes predicted, 4370 were protein coding genes, and 100 RNAs; 302 pseudogenes were also identified. The majority of the protein-coding genes (65.6%) were assigned with a putative function while the remaining ones were annotated as hypothetical proteins. The distribution of genes into COGs functional categories is presented in Table 4.

**Table 3:** Genome Statistics

**Attribute**	**Value**	**% of Total**
Genome size (bp)	4,545,624	100.00%
DNA coding region (bp)	3,870,017	85.14%
DNA G+C content (bp)	1,888,927	41.55%
Number of replicons	1	
Extrachromosomal elements	0	
Total genes	4470	100.00%
RNA genes	100	2.24%
rRNA operons	10	
Protein-coding genes	4370	97.76%
Pseudo genes	302	6.76%
Genes with function prediction	2932	65.59%
Genes in paralog clusters	1045	23.38%
Genes assigned to COGs	2702	60.45%
Genes assigned Pfam domains	2814	62.95%
Genes with signal peptides	701	15.68%
Genes with transmembrane helices	791	17.70%
CRISPR repeats	11	

**Table 4:** Number of genes associated with the general COG functional categories

**Code**	**Value**	**% age**	**Description**
J	151	3.5	Translation, ribosomal structure and biogenesis
A	0	0.0	RNA processing and modification
K	224	5.1	Transcription
L	332	7.6	Replication, recombination and repair
B	1	0.0	Chromatin structure and dynamics
D	51	1.2	Cell cycle control, mitosis and meiosis
Y	0	0.0	Nuclear structure
V	65	1.5	Defense mechanisms
T	207	4.7	Signal transduction mechanisms
M	176	4.0	Cell wall/membrane biogenesis
N	96	2.2	Cell motility
Z	1	0.0	Cytoskeleton
W	0	0.0	Extracellular structures
U	76	1.7	Intracellular trafficking and secretion
O	87	2.0	Posttranslational modification, protein turnover, chaperones
C	180	4.1	Energy production and conversion
G	104	2.4	Carbohydrate transport and metabolism
E	219	5.0	Amino acid transport and metabolism
F	66	1.5	Nucleotide transport and metabolism
H	143	3.3	Coenzyme transport and metabolism
I	48	1.1	Lipid transport and metabolism
P	139	3.2	Inorganic ion transport and metabolism
Q	28	0.6	Secondary metabolites biosynthesis, transport and catabolism
R	316	7.2	General function prediction only
S	267	6.1	Function unknown
-	1768	40.5	Not in COGs

## Insights from the genome sequence

### Heterotrophic substrate utilization

It has been shown that in *D. acetoxidans* acetate is oxidized to CO_2_ *via* the acetyl-CoA/carbon monoxide dehydrogenase (CODH) pathway [30] and all necessary genes required for this pathway have been annotated in the finished genome sequence. Interestingly, a core set of genes that is specific for this pathway shows the same arrangement in *D. acetoxidans* (Dtox_1269 to 1276) as in the distantly related homoacetogenic bacterium *Moorella thermoacetica* (*cooC/acsE*) [31]. A cluster of genes (Dtox_1697 to 1703) that is probably required for growth with butyrate as substrate could be also identified. It has been reported that acetate accumulates upon growth on butyrate and that acetate is only further metabolized to CO_2_ under conditions of carbon substrate limitation [2]. This could indicate that the acetyl-CoA/CODH pathway in this strain is only induced under conditions of energy limitation. For the utilization of primary alcohols several putative alcohol dehydrogenases may be used that are encoded at various sites in the genome of *D. acetoxidans*.

Strain 5575^T^ exhibits a prolonged lag phase upon transfer from media with acetate or butyrate as carbon source to media supplied with other organic substrates [2]. Hence, the identification of additional growth substrates might have been hampered by requiring elongated incubation times of several weeks. This could explain why the utilization of lactate is discussed controversially in the literature. According to Widdel and Pfennig [1,2] this strain is unable to use lactate, whereas Pawłowska-Ćwięk and Pado have repeatedly postulated growth on lactate as sole carbon source [32]. The genome here reported encodes a putative D-lactate dehydrogenase gene (Dtox_0988), which is however only distantly related to genes encoding enzymes known to be involved in the respiration or fermentation of lactate. Hence, it is unclear if this enzyme could be involved in the utilization of lactate by *D. acetoxidans*. In our experiments strain 5575^T^ did not show any visible growth on lactate after an incubation period of four weeks.

It was also stated that glucose, fructose, maltose and cellobiose are unsuitable electron donors for this species [2]. However, all necessary genes encoding enzymes of the Embden-Meyerhof-Parnas pathway for the conversion of sugars to pyruvate (glycolysis) were identified in the genome sequence. Hence, it is possible that this pathway is used only for the internal metabolism of carbohydrates and that a transport system for the efficient uptake of sugars is not expressed.

### Autotrophic growth

Londry and Des Marais [17] reported that *D. acetoxidans* can grow autotrophically with H_2_ as the electron donor and CO_2_ as carbon source, which is contradictory to the original species description [1,2]. Genes for several subunits of a putative Fe-only hydrogenase (Dtox_0168, Dtox_0169 and Dtox_0172 to 0178) and a [NiFe]-hydrogenase (Dtox_0791 to 0796) were detected in the genome of *D. acetoxidans*, which would confirm the finding of H_2_ utilization in this species.

The acetyl-CoA/CODH pathway is fully reversible and it was shown that in *D. autotrophicum* it is used for both the cleavage and formation of acetyl-CoA [33]. Hence, Londry and Des Marais [17] proposed that *D. acetoxidans* uses the acetyl-CoA/CODH pathway for the fixation of CO_2_ under lithoautotrophic growth conditions. They reported that during growth on H_2_/CO_2_ and sulfate, small amounts of acetate were excreted, which would confirm that the reductive acetyl-CoA pathway is used as mechanism for CO_2_ assimilation.

Interestingly, a cluster of nitrogenase genes (Dtox_1023 to 1030) could be detected within the annotated genome sequence, so that *D. acetoxidans* likely has the capacity to use dinitrogen as nitrogen source. However, the fixation of molecular nitrogen has not been analyzed by laboratory experiments in this species so far.

### Electron transport phosphorylation

The oxidation of carbon sources by dehydrogenases leads to the formation of reduced pyridine nucleotides that are most likely reoxidized in *D. acetoxidans* by a proton-translocating NADH dehydrogenase complex, which reduces menaquinones. The structure of the NADH dehydrogenase seems to be similar to complex I in the electron transport chain of *E. coli* and mitochondria. Most genes for this complex are located in a single operon (Dtox_1205 to 1215), but genes for the subunits E, F, and G are located elsewhere in the genome and often found in close proximity to genes involved in energy metabolism. Several genes encoding heterodisulfide reductases were annotated and also found close to genes involved in electron transport. Hence, it can be assumed that besides the NADH dehydrogenase complex, heterodisulfide reductases play a role in the generation of a proton gradient, as has been previously proposed for the homoacetogenic bacterium *Moorella thermoacetica* [31]. An established proton gradient could then be utilized by an ATP synthase of the F0F1-type, which is encoded in a single gene cluster (Dtox_4164 to 4172).

Besides genes involved in sulfate reduction no other genes encoding known enzymes for alternative pathways of anaerobic respiration were detected in the genome, so that in this species the utilization of electron acceptors seems to be restricted to oxidized sulfur species for sulfate reduction and CO_2_ for the synthesis of acetyl-CoA. So far, no homoacetogenic growth of *D. acetoxidans* with H_2_ or organic compounds as electron donor could be shown, so that apparently the reduction of CO_2_ is not coupled to the generation of energy in this species.

### Defense against oxidative stress

Widdel and Pfennig reported in the original species description of *D. acetoxidans* an inhibition of growth in media that were not fully reduced [1], which would indicate a high sensitivity against oxygen. Most known sulfate-reducers tolerate exposure to oxygen for at least short intervals without any recognizable cellular damage. Aerobic respiration was identified as one principal mechanism for the detoxification of oxygen in cultures of Gram-negative sulfate-reducers [34]. Accordingly, quinol oxidases of the cytochrome *bd* type that are characterized by a high-affinity to oxygen were identified in several Gram-negative sulfate-reducers [35-38] and ‘*D. reducens*’ (this study). However, no genes encoding a potential terminal oxidase could be identified in the genome of *D. acetoxidans*. Only genes encoding enzymes representing a second line of defense against reactive oxygen species were identified, for example superoxide dismutase (Dtox_4195) and catalase (Dtox_1104).

Thus, a peculiarity of the *D. acetoxidans* metabolism could be its strategy against oxidative stress. In this species other reactions may be involved in the scavenging of oxygen, so that highly sensitive compounds within the cytoplasm are protected. A potential mechanism could be the chemical reaction of oxygen with ferrous iron or iron sulfides. It was shown that cells of *D. acetoxidans* produce hydroxamic siderophores (probably deferoxamine) and accumulate large amounts of ferrous sulfide (FeS) or pyrite (FeS_2_) in their cell wall [39]. Previously, it was demonstrated *in vitro* that freshly precipitated ferrous sulfide is highly effective in the protection of strictly anaerobic cells against oxygen [40]. On the other hand, it was reported that large amounts of sulfide (above 7 mM) produced during sulfate-reduction can inhibit growth of certain *Desulfotomaculum* species [41], so that ferrous iron could also prevent toxic effects of sulfide by precipitation at the cell exterior. Several genes encoding ferrous iron transport proteins as well as subunits of ABC-type transporters specific for the uptake of Fe^3+^ siderophores were annotated in the genome of *D. acetoxidans*, which emphasizes the importance of iron in the metabolism of this species.
